# Comparative transcriptome analysis reveals different strategies for degradation of steam-exploded sugarcane bagasse by *Aspergillus niger* and *Trichoderma reesei*

**DOI:** 10.1186/s12864-017-3857-5

**Published:** 2017-06-30

**Authors:** Gustavo Pagotto Borin, Camila Cristina Sanchez, Eliane Silva de Santana, Guilherme Keppe Zanini, Renato Augusto Corrêa dos Santos, Angélica de Oliveira Pontes, Aline Tieppo de Souza, Roberta Maria Menegaldo Tavares Soares Dal’Mas, Diego Mauricio Riaño-Pachón, Gustavo Henrique Goldman, Juliana Velasco de Castro Oliveira

**Affiliations:** 1 Laboratório Nacional de Ciência e Tecnologia do Bioetanol (CTBE), Centro Nacional de Pesquisa em Energia e Materiais (CNPEM), Av Giuseppe Maximo Scolfaro 10000, Campinas, São Paulo Caixa Postal 6170, 13083-970 Brazil; 2 Faculdade de Ciências Farmacêuticas de Ribeirão Preto, Universidade de São Paulo, Av do Café S/N, Ribeirão Preto, CEP, São Paulo, 14040-903 Brazil; 3 Current address: Laboratório de Biologia de Sistemas Regulatórios, Instituto de Química, Universidade de São Paulo, Av. Prof. Lineu Prestes, 748 - Butantã - São Paulo - SP, São Paulo, CEP 05508-000 Brazil

**Keywords:** Sugarcane bagasse, RNA-seq, *Aspergillus niger*, *Trichoderma reesei*, CAZymes, Transporters, Transcription factors

## Abstract

**Background:**

Second generation (2G) ethanol is produced by breaking down lignocellulosic biomass into fermentable sugars. In Brazil, sugarcane bagasse has been proposed as the lignocellulosic residue for this biofuel production. The enzymatic cocktails for the degradation of biomass-derived polysaccharides are mostly produced by fungi, such as *Aspergillus niger* and *Trichoderma reesei*. However, it is not yet fully understood how these microorganisms degrade plant biomass. In order to identify transcriptomic changes during steam-exploded bagasse (SEB) breakdown, we conducted a RNA-seq comparative transcriptome profiling of both fungi growing on SEB as carbon source.

**Results:**

Particular attention was focused on CAZymes, sugar transporters, transcription factors (TFs) and other proteins related to lignocellulose degradation. Although genes coding for the main enzymes involved in biomass deconstruction were expressed by both fungal strains since the beginning of the growth in SEB, significant differences were found in their expression profiles. The expression of these enzymes is mainly regulated at the transcription level, and *A. niger* and *T. reesei* also showed differences in TFs content and in their expression. Several sugar transporters that were induced in both fungal strains could be new players on biomass degradation besides their role in sugar uptake. Interestingly, our findings revealed that in both strains several genes that code for proteins of unknown function and pro-oxidant, antioxidant, and detoxification enzymes were induced during growth in SEB as carbon source, but their specific roles on lignocellulose degradation remain to be elucidated.

**Conclusions:**

This is the first report of a time-course experiment monitoring the degradation of pretreated bagasse by two important fungi using the RNA-seq technology. It was possible to identify a set of genes that might be applied in several biotechnology fields. The data suggest that these two microorganisms employ different strategies for biomass breakdown. This knowledge can be exploited for the rational design of enzymatic cocktails and 2G ethanol production improvement.

**Electronic supplementary material:**

The online version of this article (doi:10.1186/s12864-017-3857-5) contains supplementary material, which is available to authorized users.

## Background

The global demand for renewable energy has led to the search and development of new technologies for biofuel production. Biofuels are produced from plants or agricultural, industrial and domestic wastes, and their use can help to reduce the world’s dependence on oil and gasoline, being a viable alternative to fossil fuels. The main types of first generation biofuels commercially used are biodiesel, biogas and bioethanol, being the latter the most widely used in the world [1]. According to the U.S. Department of Energy, 24,570 millions of gallons of ethanol were produced in 2014, and the world's top producers were the United States and Brazil, accounting together for more than 80% of the global production (http://www.afdc.energy.gov). Although in the last 10 years Brazil has lost its leadership in ethanol production, it still has best economic model of ethanol industry due to its technology and vast amount of land available to sugarcane cultivation, a cheap feedstock [2, 3]. The Brazilian fermentation process is characterized by the use of very large tanks (0.5 to 3 million liters) and high yeast cell densities (10–15% w/v). The fermentations are carried out for a short period of 6–12 hours, reaching high alcohol concentration (7–11% v/v). At the end of the fermentation, yeast cells are harvested by centrifuging and reutilized in a next fermentation cycle, a quite peculiar trait in the Brazilian process [4].

Under these conditions, 270–280 kg of bagasse per ton of crushed sugarcane are generated as byproduct [2]. Currently, most of that bagasse is burned to generate biosteam and bioelectricity, which is in turn consumed by the distillery or refinery. However, bagasse could be used to produce second generation (2G) ethanol (or cellulosic ethanol). For this purpose, the plant biomass should be hydrolyzed to release fermentable sugars from the plant cell wall. Bagasse is composed of cellulose (38%), hemicellulose (23%), lignin (25%) and a small percentage of other compounds [5]. Currently, a hectare of sugarcane can produce about 6,000 L of ethanol, and if 50% of the bagasse generated by the distilleries was used for 2G production, it would be possible to increase this volume to 10,000 L/ha [6].

2G ethanol technology includes three stages: (*i*) a physical and/or chemical pretreatment of the plant biomass, that aims to decrease its recalcitrance, improving enzyme accessibility to cellulose and recovering monosaccharides from hemicelluloses, e.g., xylose; (*ii*) saccharification, the breakdown of complex polysaccharides (mainly cellulose, but also xylose-based polysaccharides) into monosaccharides, and (*iii*) fermentation, the bioethanol production from the released monosaccharides in (*i*) and (*ii*). Although several improvements have been obtained in all these steps, the lack of yeasts capable of efficiently fermenting xylose and the high cost of the necessary enzymes in step (*ii*), represent bottlenecks to the 2G ethanol economic viability [7, 8].

Estimates of the costs of the enzymatic cocktails required for the saccharification process differ significantly, from $0.10/gal to $0.40/gal of ethanol [9]. Among other factors, these values depend on the type of biomass to be hydrolyzed, the pretreatment used, and the microorganisms that produce plant cell wall degrading enzymes (PCWDEs). In nature, the breakdown of lignocellulosic biomass is driven by a large community of microorganisms, with special highlight to the saprophytic fungi, such as *Trichoderma reesei* and *Aspergillus niger,* whose hydrolytic and oxidative enzymatic arsenal have been widely employed in these commercial enzymatic cocktails [10, 11]. The lineages leading to *T. reesei* (Sordariomycetes) and *A. niger* (Eurotiomycetes) diverged more than 310 million years ago [12], so they have been exploring for a long time different strategies to extract carbon sources required for their growth from available biomass.

*T. reesei* was isolated from the Solomon Islands during the Second World War, colonizing tents and parachutes from the U.S. army [13]. It was found that the new organism (originally named *T. viridae*) was a good producer of cellulases and, as the interest in renewable energy sources was increasing, it became the subject of several studies related to polysaccharide degradation. In this sense, the development of a derived strain through a series of induced mutagenesis experiments resulted in the hypercellulolytic RUT-C30 that is catabolite-derepressed [14, 15]. Furthermore, due to its great ability to produce and secrete large amounts of cellulases and hemicellulases, *T. reesei* is considered a model for cellulose degradation [16, 17].

*A. niger* is the most important industrial fungus in the genus *Aspergillus*, being used to produce a wide range of commercial compounds [18]. The most important is citric acid, the main acidulant used in food and beverage industries. In addition to its importance in pharmaceutical and cosmetics industries, *A. niger* has been explored to produce organic acids such as gluconic and fumaric acids [19, 20]. In relation to enzymes, *A. niger* is employed to produce pectinases, amylases and proteases, which are used in a variety of industrial processes, including juice clarification and detergent formulations [21–23]. Regarding the 2G ethanol industry, *A. niger* is used as source of enzymes for biomass degradation and host for heterologous protein production [24, 25].

The enzymes necessary for the breakdown of polysaccharides are usually subject to carbon catabolite repression (CCR), which means that growth on glucose or other preferred carbon sources represses their synthesis [26]. This process is regulated at the transcriptional level, and one of the major CCR players is the transcription factor CreA. In *T. reesei* RUT-C30, its orthologue Cre1, is truncated [14], allowing a higher enzyme production by this strain. Antagonistically, the crucial player in the induction of cellulases and hemicellulases is the transcription factor XlnR, and its orthologue in *T. reesei* Xyr1. In addition, other proteins are involved in the positive (Ace2, Ace3, Hap2/3/5, Lae1, GalX, ManR, AraR) and negative (Ace1, Cre2) regulation of gene expression of those enzymes [27–29]. However, a complete understanding of transcriptional regulation and production of PCWDEs is still lacking and needs to be better investigated.

The activation and repression of these regulators depend on the inducers present in the environment or culture media, such as cellobiose, xylobiose, xylose, lactose, and sophorose, each of them being species-dependent [28] and responsible to trigger the production of the enzymes that degrade biomass. These enzymes (and other proteins involved in the assembly of complex carbohydrates) are collectively identified as Carbohydrate-Active enZymes (CAZymes) [30]. They are classified in classes and families, according to their sequence, structure and molecular mechanisms, by the online database CAZy (http://www.cazy.org). Enzyme classes covered by CAZy include glycoside hydrolase (GH), carbohydrate esterase (CE), polysaccharide lyase (PL), glycosyltransferases (GT), and enzymes with auxiliary activity (AA). All of these classes contain enzymes that act on the biomass deconstruction, with the exception of GT and CE family 10.

Thus, gaining detailed knowledge about the molecular mechanisms behind biomass degradation and enzyme production can enable a fine genetic manipulation of these strains in order to increase their hydrolytic potential or the discovery of new enzymes or proteins, and their combinations that can improve the saccharification process. Although several studies have been done on *A. niger* and *T. reesei* to understand their transcriptional responses when grown on lignocellulosic biomass, we have studied for the first time, their time-dependent transcriptional response when grown on pretreated bagasse, one of the largest and most inexpensive lignocellulosic ethanol feedstocks.

## Results and discussion

Simple sugars do not induce the full enzymatic repertoire that is encoded by a fungal genome. Moreover, the enzymatic arsenal produced in response to a specific plant biomass is likely to vary towards different substrates. Understanding the molecular mechanisms, particularly transcriptional control, in fungi that have evolved to degrade vegetal biomass will provide clues to improve the biomass saccharification step, thus being of paramount importance for the economic viability of 2G biofuel production.

There are few studies analyzing the transcriptome of *A. niger* grown in plant biomass (Table 1). Pullan et al. [31] and Delmas et al. [32] described a comparative transcriptional study of this fungus grown on willow and wheat straw, respectively. Van Muster et al. [33] investigated the induction of CAZymes in wheat straw and in a carbon starvation condition. De Souza et al. [34] analyzed the transcriptome of *A. niger* grown in sugarcane bagasse, using a microarray platform. Besides these works, there are some studies using monomeric sugars as carbon sources [35–37], under starvation [38] or using mutant strains [39]. In *T. reesei,* two studies described its global gene expression profile growing on wheat straw [40] and wheat straw in comparison to lactose [41]. Dos Santos Castro et al. [42] compared the transcriptome of *T. reesei* during growth in cellulose, sophorose, and glucose; Hakkinen et al. [43] investigated the *T. reesei* transcriptome in several substrates, including sugarcane bagasse, using a microarray platform (Table 1). Two publications compared the secretome of both fungi, using proteomics, growing on complex substrates [44, 45]. In this work we studied, for the first time, to the best of our knowledge, the transcriptional response of both fungi when grown on pretreated lignocellulosic biomass.

**Table 1:** *T. reesei* RUT-C30 and *A. niger *N402 transcriptome studies using different carbon sources and growth conditions

Species	Carbon source	Pre-treatment	Time-course	Technology	Total number of CAZymes genes upregulated	Reference
*T. reesei* QM9414	Cellulose, sophorose and glucose	-	24, 48 and 72 h, cellulose; 24 and 48 h, glucose; 2, 4 and 6 h, sophorose	RNA-seq (Illumina HiSeq 2000)	21, sophorose; 25, cellulose; 17, glucose^a^	42
*T. reesei* NG14 and RUT-C30	Lactose and glucose	-	1, 3, 6 and 24 h, microarray; 1, 6 and 24 h, RNA-Seq	Microarray and RNA-seq (Illumina HiSeq 2000)	29, *T. reesei* NG 14; 26, *T. reesei* RUT C30^b^	46
*T. reesei* QM6a	Wheat straw and glucose (control)	Ball milling	Wheat straw 24 h vs. glucose 48 h	RNA-seq (SOLiD 4)	33, wheat straw^c^	40
*T. reesei* QM9414	Wheat straw, lactose and glucose (control)	Grinding and acid thermochemical	50 h, wheat straw; 28 h, lactose	Microarray	40, wheat straw; 7, lactose;	41
*T. reesei* RUT-C30	Ground bagasse (BO); steam-exploded bagasse (BS); enzymatically hydrolysed(BE); steam-exploded wheat straw(WH); steam-exploded spruce (SP); oat spelt xylan (XO); birch xylan (XB); avicel cellulose 1%(AV1); avicel cellulose 0.75% (AV0.75); sophorose (SO)	Ground (bagasse), enzymatically hydrolsed (bagasse), steam-exploded (spruce, wheat straw, bagasse)	Lignocellulosic substrate 6, 17, 41 and 65 h	Microarray	87 (BO), 98 (BS), 124 (BE), 82 (WH), 51 (SP), 68 (XO), 94 (XB), 58 (AV1), 43 (AV0.75), 57 (SO)	43
*A. niger* N402	Willow and glucose	Ball milling	Willow 24 h vs. glucose 48 h	RNA-seq (SOLiD 5500)	107, willow^d^	31
*A. niger* N402	Wheat straw	Ball milling	Glucose 48 h + wheat straw 6 and 24 h	RNA-seq (SOLiD 5500)	64, wheat straw	33
*A. niger* N402	Wheat straw and glucose (control)	Ball milling	24 h, wheat straw	RNA-seq (SOLiD 4)	191, wheat straw ^d^	32
*A. niger* N402	Sugarcane bagasse (SB) and fructose (control)	Steam-exploded	Fructose 24 h + SEB 6, 12 and 24 h	Microarray	67, SB	34

^a^Total RNA of the different time points of the same carbon source was pooled and sequenced; ^b^ It was not cited how many CAZymes were induced in RNA-seq data, thus we considered as upregulated genes with log_2_ ratio > 1 at least in one time point; ^c^ Considering only the CAZy genes with fold change > 20; ^d^ Considering only genes having fold change values > 2

We chose to compare the *T. reesei* RUT-C30 and *A. niger* N402 strains. As mentioned before, RUT-C30 is an industrial strain, which has important mutations that affect its lignocellulolytic phenotype. We were interested on *A. niger* N402 as in a previous work from our group [44], this strain showed a higher secretion of CAZymes than RUT-C30. Therefore, we wanted to gain more insights about the differences at the transcriptional level between both strains, and to identify potential new genes involved in the lignocellulose breakdown. *A. niger* and *T. reesei* were grown on steam-exploded sugarcane bagasse (SEB) as sole carbon source and biological duplicates were collected at 6, 12 and 24 h. Both fungi were also grown on fructose for 24 h as control condition in order to identify differentially expressed genes (DEG), responding to the lignocellulosic substrate. Poly-adenylated transcripts were sequenced in a HiSeq2000 system, and the resulting short reads were mapped to the respective reference genomes, achieving mapping rates of over 93% for both strains (Additional file 1). Analysis of exon-exon junctions revealed that saturation was achieved, i.e., increasing the number of sequenced reads would not have resulted in significantly more exon-exon junctions detected (data not shown). The average Pearson correlation coefficient between duplicated samples was 0.94.

**Fig. 1:**
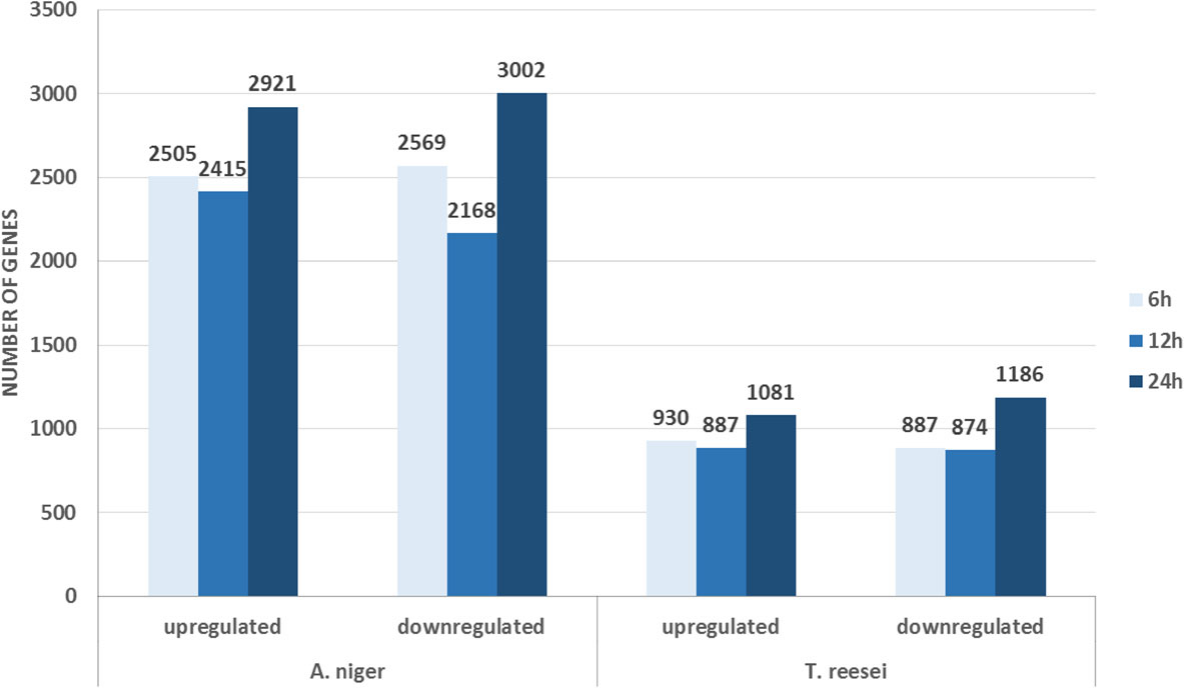
Number of genes up and downregulated in *A. niger* N402 and *T. reesei* RUT-C30 grown on SEB for 6, 12 e 24 hours (h). The full list of DEGs is shown in Additional file 2

We detected 7,359 genes in *A. niger* and 2,945 in *T. reesei* that are induced or repressed when SEB was used as the unique carbon source (genes with at least two-fold change) (Fig. 1 and Additional file 2). *A. niger* had 2.5 times more DEGs than *T. reesei,* despite both genomes being of approximately the same size (33.9 Mbp vs 32.7 Mbp), and *A. niger* only having 1.4 times more predicted protein-coding genes than *T. reesei* (14,165 vs 9,852). Thus, gene content alone cannot explain the difference in the number of DEGs between the two strains, but instead it should be a consequence of the individual ability of each fungus to respond to the recalcitrant biomass and to activate different pathways and genes for its degradation. It is worth noting that during the time course, time point 24 h showed a slightly larger number of DEGs in both fungi, probably due to the higher quantities of small molecules released after biomass cell wall degradation that could act as inducers.

**Fig. 2:**
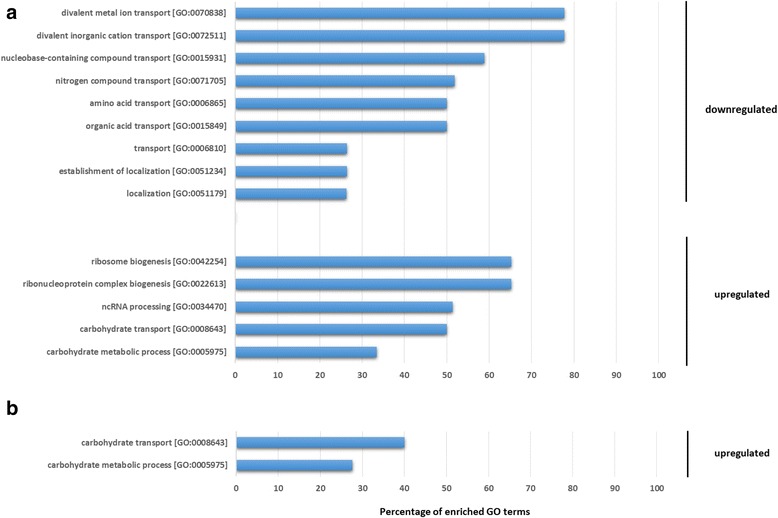
Gene ontology enrichment analysis of the differently genes expressed in the growth on SEB for 24 hours. **a** up and downregulated genes in *A. niger* N402, (**b**) upregulated genes in *T. reesei* RUT-C30. No categories were enriched for *T. reesei* RUT-C30 downregulated genes. It was only considered categories with e-value ≤ 1.0E-05

Gene Ontology (GO) enrichment analysis for the gene set induced at time point 24 h (Fig. 2, Additional file 3) showed that both *A. niger* (Fig. 2a) and *T. reesei* (Fig. 2b) had in common the terms Carbohydrate metabolic process (GO: 0005975) and Carbohydrate transport (GO: 0008643); in addition, three categories associated with gene expression processes were enriched in *A. niger*, such as ribonucleoprotein complex biogenesis (GO: 0022613), ribosome biogenesis (GO: 0042254) and ncRNA processing (GO: 0034470) (Fig. 2a). Regarding the repressed genes, *A. niger* had enriched categories associated with nitrogen metabolism, such as nitrogen compound transport (GO: 0071705) and amino acid transport (GO: 0006865) (Fig. 2a). In the set of repressed genes in *T. reesei*, no GO categories were over represented.

The two GO terms commonly enriched for both fungi in the set of induced genes in SEB at 24 h (GO: 0005975 and GO: 0008643) include genes coding for CAZymes and transporters. This leads us to speculate that *T. reesei* and *A. niger* were spending their energy to the transcription and production of cellulolytic compounds, which were probably being secreted for biomass degradation and the release of small sugars, as shown by Borin et al. [44]. The released mono and disaccharides may serve as inducers of CAZyme-coding gene expression and activators of membrane transporters, whose function is to allow the transport of sugars from the extracellular to the intracellular microenvironment and also have a role in the induction of cellulase and hemicellulose biosynthesis [46, 47]. For this reason, the protein synthesis machinery of the fungi should be activated to carry out protein translation of the induced genes, which explain the GO terms enriched in the set of *A. niger* induced genes in SEB at 24 h*.*

As our main interest is to understand how these fungal strains rearrange their transcriptional programs in response to the availability of a complex lignocellulosic substrate, we focused the following analyses on CAZymes, transporters, transcription factors, and other genes of interest. Genes coding proteins of unknown function were also of particular interest as they can represent new targets for further analysis.

### CAZymes

**Fig. 3:**
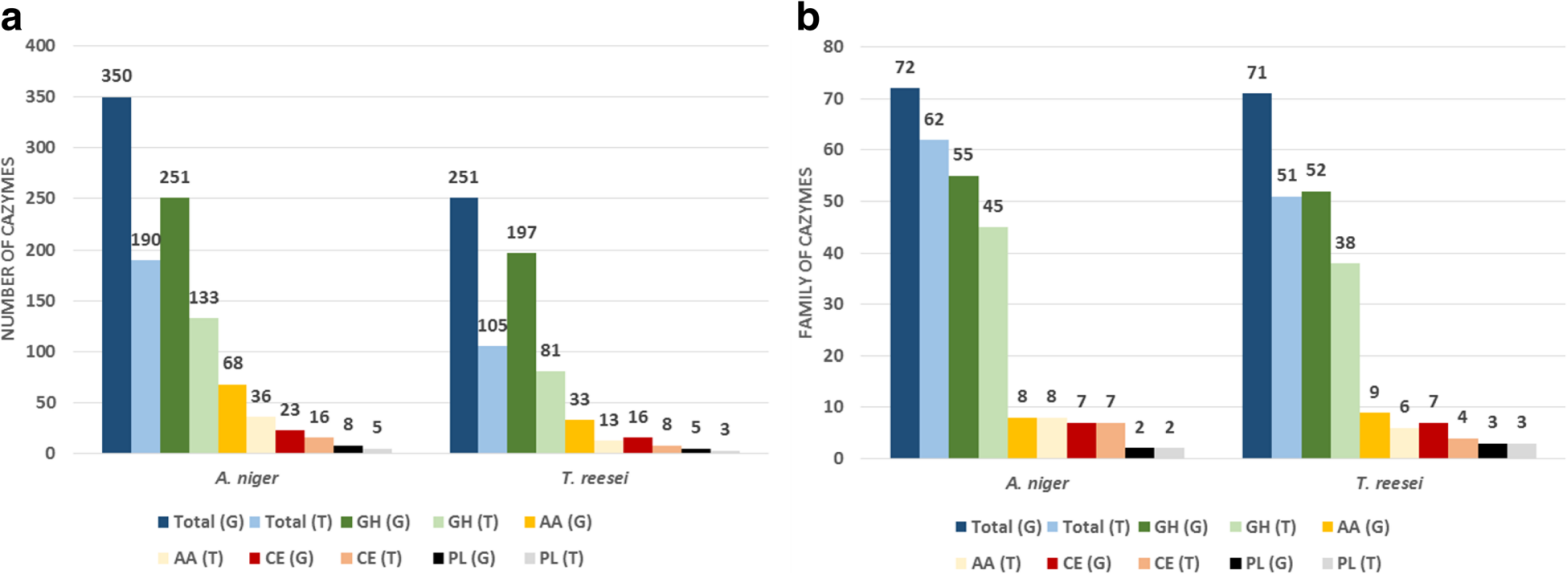
Total number of CAZymes (excluding GT and CE10) (**a**) and their respective families (**b**) found in the genomes (G) and transcriptomes of *A. niger* N402 and *T. reesei* RUT-C30 grown on SEB (T)

CAZymes are Carbohydrate-Active Enzymes classified by the CAZy database. We focused on these enzymes, excluding the classe of glycosyl transferase and the carbohydrate esterase family 10, as they are not related to carbohydrate degradation. We found that 190 CAZymes, in 62 different families, were upregulated in *A. niger*, while 105 CAZymes were upregulated in *T. reesei*, from 51 families (Fig. 3a and 3b, and Additional file 4 and 5). The genomes from both species have approximately the same proportion of CAZymes related to the total number of predicted genes, approx. 2.5% for each species. However, *A. niger* is expressing a larger proportion of CAZymes than *T. reesei* (1.3% vs 0.8%, respectively), also from a larger number of different families (Fig. 3). The CAZyme transcriptional response in *A. niger* is more diverse than *T. reesei,* when SEB is the source of carbon.

Previous papers have also assessed the transcriptional changes in these two fungi when growing on simple or complex carbon sources (Table 1). Despite differences in fungal growth and experimental design, the transcriptional changes observed could be also due to the composition of substrate utilized, as well as the severity of the pretreatment applied to biomass. We are presenting herein the first study that compares the transcriptional response of these two fungal strains when grown on SEB as sole carbon source. Steam-explosion induces changes in lignin structure, which coupled to partial hemicellulose hydrolysis, increases substantially the cellulosic fraction availability and as consequence, its hydrolysis [48]. As result, more sugars (polysaccharides, oligosaccharides and monomers) are released to the medium, what might thus induce the expression of a larger number of different CAZymes, than observed before. The transcriptional response of *A. niger* when grown on SEB was previously studied using microarray, revealing a smaller number of differentially expressed CAZymes [34] (67 genes, Table 1). This difference might result from the microarray platform having a narrower dynamic range for expression values in comparison to RNA-seq approaches [49].

Discrepancies were also found in relation to the temporality of gene expression. For *A. niger*, Delmas et al. [32] showed important genes related to biomass degradation being induced along their time series study. For instance, the cellobiohydrolase B (*cbhB*, An01g11660, GH7) and an arabinofuronosidase (*abfB*, An15g2300, GH54) were expressed after 6 h of growth in media with wheat straw, the cellobiohydrolase A (*cbhA*, An07g09330, GH7), one lytic polysaccharide monooxygenase (An12g04610, AA9) and one hydrophobin (*hsbA*, An09g00840) after 12 h, and another hydrophobin (*hfbD*, An08g09880) only after 24 h. We found these genes induced since 6 h, with high values of log_2-_fold change and expression values (expression value RPKM; Additional file 4). Curiously, the only exception is the *cbhA* that was upregulated at 6 and 12 h, but not at 24 h. Analyzing the raw data, this gene was highly expressed both in SEB and in fructose at 24 h (data not shown), but it was not differently regulated. In relation to *T. reesei*, Poggi et al. [50] showed a similar expression of the main CAZymes (*cel5a*, *cel12a*, *cel45a*, *cel74a*) during a time course (1, 3, 6 and 24 h) using lactose as inducer of cellulolytic gene expression. Some differences, such as the absence of *cel7b* in the latest time point in lactose, could be a signal saturation problem present in microarray analysis.

In addition to the disparities in the total number of differentially expressed CAZymes between *A. niger* and *T. reesei*, there are also important differences in the families that were being regulated (Additional file 4 and 5). Genes exclusively found induced in *A. niger* were from the families CE8 (An02g12505 and An04g09690), CE12 (An04g09360, An05g02270 and An09g02160), PL1 (An10g00870, An11g04030, An14g04370 and An19g00270), PL4 (An14g01130), that play distinct roles in the breakdown of pectin, and GH51 (α-L-arabinofuranosidase) that hydrolyzes the glycosidic bond between L-arabinofuranosides side chains of hemicelluloses such as arabinoxylan (Table 2). Conversely, for *T. reesei*, the following families were exclusively induced in this microorganism: the GH45 endoglucanase (25940) that acts on the hydrolysis of soluble β-1,4 glucans, a CE15 glucuronoyl esterase (125575) and a GH115 xylan-α-glucuronidase (25542) that can cleave glucuronic acid residues from xylan backbone (Table 3).

**Table 2:** The main CAZymes related to biomass deconstruction upregulated in *A. niger* N402 transcriptome

ID AspGD^a^	Description^a^	Gene name	CAZy Family	Peptide Signal^b^	log_2_FC^c^			Predicted substrate
					6h	12h	24h	
An16g00060	Hypothetical protein	-	CE1	N	3.32	3.09	4.91	Unknown
An08g05230	Lytic polysaccharide monooxygenase	*-*	AA9	Y	2.24	1.55	-	Cellulose
An12g02540	Lytic polysaccharide monooxygenase	*-*	AA9	Y	5.41	6.57	3.30	Cellulose
An12g04610	Lytic polysaccharide monooxygenase	*-*	AA9	Y	8.49	8.35	4.82	Cellulose
An14g02670	Lytic polysaccharide monooxygenase	*-*	AA9, CBM1	Y	1.71	-	-	Cellulose
An15g04900	Lytic polysaccharide monooxygenase	*-*	AA9, CBM1	Y	2.16	1.80	-	Cellulose
An09g00120	Feruloyl esterase	*faeA*	CE1	Y	7.80	6.93	5.95	Arabinoxylan, pectin
An12g05010	Acetyl xylan esterase	*axeA*	CE1	Y	8.22	6.63	5.68	Xylan
An12g02550	Feruloyl esterase	*-*	CE1	Y	8.15	7.38	5.12	Xylan, pectin
An07g03100	Putative esterase E	*-*	CE1	N	1.04	-	-	Unknown
An07g08940	Cellulose-binding GDSL lipase/acylhydrolase	*-*	CE16	Y	6.73	5.21	3.94	Xylan, mannan
An14g02170	Cutinase 1	*-*	CE5	Y	2.97	2.99	1.34	Cutin
An11g00110	Cutinase 2	*-*	CE5	Y	1.84	1.28	1.72	Cutin
An03g03740	Β-glucosidase	*bgl4*	GH1	N	2.82	1.87	-	Cellulose
An04g03170	Β-glucosidase	*-*	GH1	Y	6.95	4.53	5.27	Cellulose
An11g02100	Β-glucosidase	*-*	GH1	Y	6.30	5.44	4.86	Cellulose
An03g00940	Endoxylanase	*xynA*	GH10	Y	7.90	6.38	5.00	Xylan
An01g00780	Endoxylanase	*xynB*	GH11	Y	9.42	7.71	6.21	Xylan
An15g04550	Endoxylanase	*xynV*	GH11	Y	4.15	2.40	1.73	Xylan
An14g02760	Endoglucanase	*eglA*	GH12	Y	9.02	7.58	4.78	Cellulose
An01g03340	Xyloglucan active endoglucanase	*-*	GH12	Y	5.48	4.43	3.20	Cellulose
An11g06080	Β-glucosidase	*-*	GH3	N	3.95	3.19	1.90	Cellulose
An11g06090	Candidate β-glucosidase	*-*	GH3	Y	3.76	3.34	2.20	Cellulose
An02g07590	Candidate β-glycosidase related to β-N-acetylhexosaminidase	*-*	GH3	N	-	-	1.01	Unknown
An07g07630	Β-glucosidase	*-*	GH3	Y	6.32	5.45	5.02	Starch
An11g00200	Β-glucosidase	*-*	GH3	Y	7.44	6.38	6.21	Cellulose
An14g01770	Β-glucosidase	*-*	GH3	Y	1.78	1.24	-	Cellulose
An15g04800	Β-glucosidase	*-*	GH3	Y	1.48	-	1.73	Cellulose
An17g00520	Β-glucosidase	*-*	GH3	N	5.69	4.43	1.67	Cellulose
An18g03570	Β-glucosidase	*bglA*	GH3	Y	6.38	5.72	4.60	Cellulose
An01g09960	Β-xylosidase	*xlnD*	GH3	Y	8.24	6.05	4.47	Xylan, pectin
An17g00300	Β-xylosidase/α-arabinofuranosidase	*xarB*	GH3	Y	6.10	4.39	3.49	Xylan, pectin
An02g10550	Endoarabinanase	*abnC*	GH43	Y	5.63	5.26	5.09	Pectin
An09g01190	Endoarabinanase	*abnA*	GH43	Y	5.14	4.47	2.93	Pectin
An15g03550	Endoarabinanase	*-*	GH43	Y	8.24	7.84	6.12	Pectin
An08g01900	Β xylosidase	*-*	GH43	Y	5.43	4.17	4.71	Xylan
An02g00140	Β-xylosidase	*-*	GH43	N	4.41	3.39	4.14	Xylan
An11g03120	Β-xylosidase	*xynD*	GH43	Y	3.55	3.20	2.56	Xylan, pectin
An18g03330	Exo-1,3-galactanase	*-*	GH5	N	1.17	1.38	-	β-1,3-Glucan
An07g08950	Endoglucanase	*eglC*	GH5	Y	5.46	4.22	2.19	Cellulose
An06g02060	Exo-1,3-galactanase	*-*	GH5	Y	1.18	-	1.35	Cellulose
An18g04100	Exo-1,3-galactanase	*exgA*	GH5	Y	4.46	5.19	5.08	β-1,3-Glucan
An03g01050	Exo-1,6-galactanase	*-*	GH5	Y	2.93	-	-	Cellulose
An01g11670	Endoglucanase	*-*	GH5, CBM1	Y	8.00	6.48	4.22	Cellulose
An08g01710	α- arabinofuranosidase	*abfC*	GH51	N	4.61	3.73	3.91	Xyloglucan, xylan, pectin
An15g02300	α-arabinofuranosidase	*abfB*	GH54, CBM42	Y	6.04	3.81	1.03	Xyloglucan, xylan, pectin
An08g01760	Cellobiohydrolase	*-*	GH6	Y	9.03	7.97	5.43	Cellulose
An12g02220	Cellobiohydrolase	*-*	GH6, CBM1	Y	11.59	10.76	7.40	Cellulose
An03g00960	Arabinoxylan arabinofuranohydrolase	*axhA*	GH62	N	7.52	6.18	4.95	Xylan
An14g05800	α-glucuronidase	*aguA*	GH67	Y	7.51	5.50	4.83	Xylan
An07g09330	Cellobiohydrolase	*cbhA*	GH7	Y	4.36	4.71	-	Cellulose
An01g11660	Cellobiohydrolase	*cbhB*	GH7, CBM1	Y	8.45	6.69	3.08	Cellulose
An01g01870	Xyloglucan active endoglucanase	*eglC*	GH74, CBM1	Y	1.67	1.67	-	Cellulose

^a^The *Aspergillus* Genome Database was used to recover the *A. niger* proteins ID and description; ^b^ The presence of signal peptide was predicted by SignalP 4.1 Server; ^c^ Log_2_ fold change

**Table 3:** The main CAZymes related to biomass deconstruction upregulated in *T. reesei* RUT-C30 transcriptome

ID JGI*	Description^a^	Gene name	CAZy Family	Peptide Signal^b^	log_2_FC^c^			Predicted substrate
					6h	12h	24h	
122518	Lytic polysaccharide monooxygenase	*cel61b*	AA9	Y	3.23	6.54	9.09	Cellulose and β-glucan
139633	Lytic polysaccharide monooxygenase	*cel61a, egl4*	AA9, CBM1	Y	3.84	6.10	8.59	Cellulose and β-glucan
136770	Acetyl esterase	*aes1*	CE16	Y	6.33	7.50	7.74	Xylan
5643	Candidate acetyl esterase	*-*	CE16	Y	1.30	1.45	1.07	Xylan
128705	Candidate acetyl xylan esterase	*-*	CE3	N	1.60	1.82	1.55	Xylan
35989	Candidate acetyl xylan esterase	*-*	CE3	N	-	-	1.34	Xylan
134377	Candidate acetyl xylan esterase	*-*	CE3	N	2.82	3.01	3.00	Xylan
88887	Candidate acetyl xylan esterase	*-*	CE5	N	4.83	6.01	8.93	Xylan
139631	Acetyl xylan esterase	*axe1*	CE5, CBM1	Y	4.44	6.46	9.12	Xylan
127115	β-glucosidase	*bgl2, cel1a*	GH1	N	3.78	4.57	5.96	Cellulose and β-glucan
77989	Candidate β-glucosidase	*cel1b*	GH1	N	1.78	2.42	2.83	Cellulose and β-glucan
23616	Endo-β-1,4-xylanase	*xyn3*	GH10	Y	2.69	4.78	8.53	Xylan
124931	Endo-β-1,4-xylanase	*xyn2*	GH11	Y	6.38	7.56	8.91	Xylan
134945	Candidate endo-β-1,4-xylanase	*xyn5*	GH11	N	5.54	6.06	6.80	Xylan
38418	Endo-β-1,4-xylanase	*xyn1*	GH11	Y	2.01	3.00	2.73	Xylan
25542	Candidate xylan-α-1,2-glucuronidase/α-(4-O-methyl)-glucuronidase	*-*	GH115	N	-	1.11	-	Xylan
124438	Endo-β-1,4-glucanase	*egl3, cel12a*	GH12	Y	1.74	5.38	8.96	Cellulose and β-glucan
140746	β-xylosidase	*bxl1*	GH3	Y	7.84	7.57	8.25	Xylan
25095	Candidate β-glucosidase	*cel3b*	GH3	Y	-	-	1.10	Cellulose and β-glucan
125268	Candidate β-glucosidase	*cel3c*	GH3	N	2.35	3.21	4.31	Cellulose and β-glucan
136547	β-glucosidase	*bgl1/cel3a*	GH3	N	1.75	3.61	7.14	Cellulose and β-glucan
122639	Candidate β-glucosidase	*cel3d*	GH3	N	3.23	4.17	5.76	Cellulose and β-glucan
74305	Candidate β-glucosidase	*cel3e*	GH3	Y	1.31	1.36	1.51	Cellulose and β-glucan
82126	Cand. β-glucosidase/glucan 1,4-β-glucosidase	*bgl3f*	GH3	Y	2.52	3.41	5.15	Cellulose and β-glucan
74129	Candidate β-xylosidase	*xyl3b*	GH3	N	2.12	2.70	1.99	Xylan
77521	Candidate β-xylosidase/α-L-arabinofuranosidase	*-*	GH43	N	4.21	4.76	5.31	Others
25940	Endo-β-1,4-glucanase	*egl5, cel45a*	GH45, CBM1	Y	2.95	5.41	8.52	Cellulose and β-glucan
72489	Endo-β-1,4-glucanase	*egl2, cel5a*	GH5, CBM1	Y	3.27	5.74	8.57	Cellulose and β-glucan
122377	β-Mannanase	*man1*	GH5, CBM1	Y	2.52	4.86	8.77	Mannan and galactomannan
102517	α-L-arabinofuranosidase I	*abf1*	GH54, CBM42	Y	2.55	1.51	-	Xylan
122470	Cellobiohydrolase	*cbh2, cel6a*	GH6, CBM1	Y	2.73	5.28	8.24	Cellulose and β-glucan
118070	Candidate α-L-arabinofuranosidase	*abf2*	GH62	Y	5.88	4.90	7.03	Xylan
90302	α-Glucuronidase	*glr1*	GH67	Y	6.96	7.33	7.62	Xylan
125125	Cellobiohydrolase	*cbh1, cel7a*	GH7, CBM1	Y	3.49	5.84	8.75	Cellulose and β-glucan
5304	Endo-β-1,4-glucanase	*egl1, cel7b*	GH7, CBM1	Y	2.63	5.05	8.39	Cellulose and β-glucan
111943	Xyloglucanase	*cel74a*	GH74, CBM1	Y	2.04	2.18	3.87	Xyloglucan

^a^ *Trichoderma reesei* RUT C30 v1.0 database from JGI was used to recover the *T. reesei* proteins ID and description; ^b^ The presence of signal peptide was predicted by SignalP 4.1 Server; ^c^ Log_2_ fold change

**Fig. 4:**
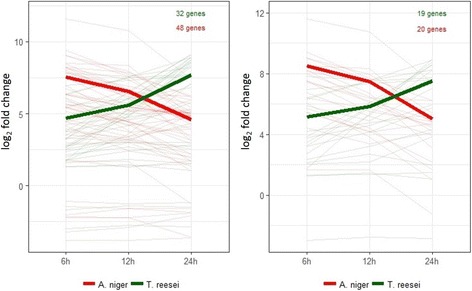
Expression profile of the genes belonging to the main CAZyme families related to biomass deconstruction in *A. niger* N402 and *T. reesei* RUT-C30. A) Average of log_2_(fold changes) from all genes listed in Table 1 and Table 2. B) Average of log_2_(fold changes) of the predicted orthologues genes (listed in Additional file 6). Thinner lines represent the individual gene expression (log_2_(fold change)) for each fungus and the thicker ones the average of these values. Only genes upregulated in all time points (6, 12 and 24 h) were considered

We were then interested in understanding the general expression profile of the CAZyme families related to biomass degradation (AA9, CE1, CE3, CE5, CE16, GH1, GH3, GH5, GH6, GH7, GH10, GH11, GH12, GH43, GH45, GH51, GH54, GH62, GH67, GH74, GH93, GH115 [51]). It could be observed that the two strains behaved in opposite ways: *A. niger* strongly induced the biomass degrading genes at the beginning (6 h) of the growth in SEB, and their expression decreased over time, while *T. reesei* had an increasing gene expression during the time course (Fig. 4a).

As the number of genes induced by each microorganism is different, we decided to focus on the candidate orthologues for these CAZymes (Additional file 6) and observed the same general trend (Fig. 4b). This trend was confirmed when looking at the individual CAZyme families (Additional file 7). This was further confirmed by RT-qPCR for some genes that encode essential enzymes to biomass degradation (Additional file 8 and 9), with significant Pearson correlations (> 0.95 for the most genes). It is interesting that CAZymes from *A. niger* showed this decreasing expression from 6 to 24 h. This gene profile was already reported by Souza et al. [34] and might represent a standard mechanism in this strain to act upon contact with complex biomass sources, and a different control in the expression of these enzymes in relation to *T. reesei* strain*.*

When looking at the 20 most strongly induced genes in *T. reesei*, it was noticeable that since the early time point (6 h), the majority of them correspond to CAZymes important to the carbon degradation, such as GH10, GH11, CE1, GH6, GH62 and others (Additional file 10). The expression of most of them increased during the time course. However, curiously, the transcriptional response of *A. niger* grown on bagasse was dramatically different; the CAZymes were prevalent only in the earliest time point and, as showed above for the main families, their expression decreased during the time course (Additional file 11, 7, 8). Despite the prevalence of CAZymes among the 20 most strongly induced genes in *T. reesei*, the average expression level was two times higher in *A. niger* at the time points 6 and 12 h (Additional file 11). We also noticed that *A. niger* N402 and *T. reesei* RUT-C30 induced a central set of shared genes coding for the most important CAZymes (such as β-glucosidases, cellobiohydrolases, endoglucanases, xylanases, β-xylosidases, lytic polysaccharide monooxygenases (LPMOs)) that are activated by a variety of lignocellulosic substrates, but have different strategies to degrade biomass.

Among the main CAZymes, AA enzymes have been the focus of several recent studies, with special highlight to the LPMOs (AA9, AA10, AA11 and AA13), as they can be involved in the oxidative breakdown of cellulose, hemicellulose and lignin [52], as well as in the biotransformation or detoxification of lignocellulosic compounds [53]. It is important to note here that *A. niger* has a considerable larger number of AAs compared to *T. reesei* (68 vs 33), showing the largest difference of all CAZymes between the two strains. Few studies have been published for AAs in these saprophytic fungi. Only AA9 (former GH61) was characterized in *T. reesei* [54–56], and two glucose oxidase (AA3) have been studied in *A. niger* [57–59]. Nevertheless, our data suggest they might have an important role on biomass degradation since 53% e 39% of all AAs found in *A. niger* and *T. reesei* genomes, respectively, were induced by growth in SEB in both strains.

Five AA9-coding genes (An08g05230, An12g02540, An12g04610, An14g02670 and An15g04900) were upregulated in *A. niger* and they had their highest expression at 6 h (Table 2 and Additional file 4), with exception of one gene (An12g04610), which expression decreased during the time course. In contrast, the two AA9 upregulated genes of *T. reesei* (139633 and 122518) showed an increasing expression during the later periods of the time course, reaching notable fold change values (386 and 543 times, respectively) at 24 h (Table 3 and Additional file 5). We could predict orthologues of the *T. reesei* 139633 gene in *A. niger*, genes An12g04610 and An14g02670, with both of these presenting different expression profiles (Additional file 6). As mentioned before, these differences were also observed to the gene expression profile of other CAZymes (GHs and CEs) in both fungal strains (Fig. 4a).

LPMOs have three different electron transfer systems [60]. The systems 1 and 2 reduce indirectly the active site of LPMOs by transferring electrons through the cytochrome domain of cellobiose dehydrogenase (CDH; AA3.1) or by fungal and plant-derived LPMO-reducing phenols, respectively. The third system is comprised of GMC oxidoreductases (Glucose-Methanol-Choline oxidoreductases; AA3.2 and AA3.3) that regenerate those phenolic compounds to reduce the LPMO [60]. *A. niger* has one gene coding for a putative CDH (An02g09270), however it was not found induced in the conditions assayed here; moreover, *T. reesei* genome does not have a *cdh* gene. Conversely, several genes coding AA3.2 and AA3.3 (alcohol oxidase, aryl-alcohol oxidase, glucose oxidase, Additional file 4 and 5) were induced by SEB in both fungi. These oxidative enzymes are responsible for hydrogen peroxide and oxygen radicals formation, that are reactive molecules that can attack the polysaccharides and lignin components of lignocellulose [61]. Then, although the oxidative degradation of lignocellulose has not been investigated, our data allows to hypothesize that both fungal strains could employ enzymatic and non-enzymatic redox reactions to break down the lignocellulose.

Beyond the annotated CAZymes, 15 upregulated genes were found with significant CAZy domains (e-value < 1.00E-17 and coverage > 0.45) in *A. niger* (12 AA, 2 CE e 1 GH) and only 2 to *T. reesei* (2 AA) (Additional file 12). Most of these genes were expressed at all time points analyzed; however, whether these proteins represent new CAZymes, still needs to be investigated.

### Transporters

**Fig. 5:**
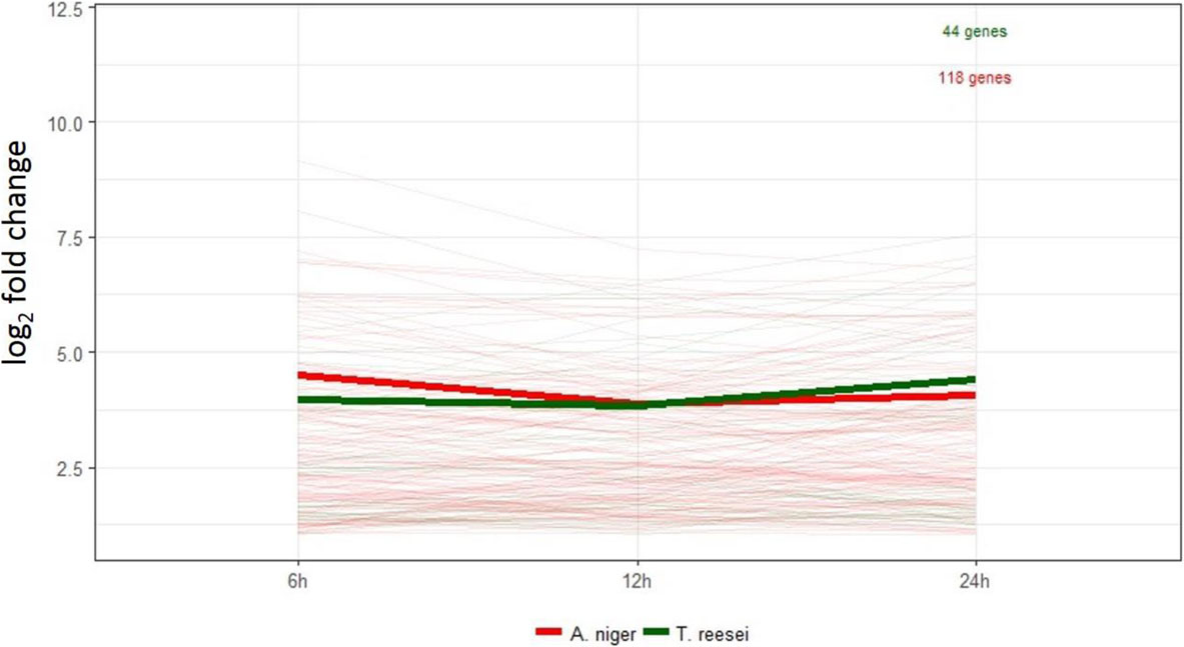
Expression profile of genes encoding transporters in *A. niger* N402 and *T. reesei* RUT-C30 transcriptomes. Average of log_2_(fold change) from all genes listed in Additional file 13 and 14. Thinner lines represent the individual gene expression (log_2_(fold change)) for each fungus and the thicker ones the average of these values. Only genes upregulated in all time points (6, 12 and 24 h) were considered

Both fungi have a similar number of transporters: the genome of *A. niger* has 865 transporter-coding genes [62] and *T. reesei* has 816 [63]. In *A. niger* 174 genes annotated as sugar transporters or without predicted substrate were upregulated (Additional file 13). About half of them were strongly (five to ten times) induced in SEB at all time points. In *T. reesei* 83 transporters were induced in SEB (Additional file 14). The expression profile during the time course was similar between both fungi (Fig. 5a), the fold changes decreased at 12 h, increasing at 24 h, which clearly contrasts with the expression profiles of CAZymes related to biomass deconstruction (Fig. 4). Although some sugar transporters are known to be regulated by transcription factors that regulate CAZymes as well [34, 64], the observed differences between the expression profiles of transporters and CAZymes suggest that different set of regulators might be controlling the expression of these genes.

These transporters are proteins to be studied in relation to their potential of transport sugars released from vegetal biomass, such as xylose, cellobiose, and arabinose and can be used to the genetic engineering of industrial strain applied in different industrial fermentations [65, 66]. Recently, Sloothaak et al. [67] applied a pipeline to identify putative xylose transporters to the *in silico* deduced proteomes of *T. reesei* and *A. niger*. From the top 15 highest scoring proteins found in each genome, only four genes were found as being upregulated in *T. reesei*, including *str1* (138519) and *str2* (136712) that were characterized as xylose transporters. In relation to *A. niger*, eight putative xylose transporter genes were found upregulated, including *xltB* (An11g05280). The product of *xltB* transports xylose with the highest specificity described so far [67].

Additionally, recently some transporters have been reported to be involved in the induction of CAZymes [46, 47, 68]. For instance, *crt1* (109243) and *str1* (138519) transporters-coding genes of *T. reesei* were strongly activated by SEB at all time points (Additional file 14), with special attention to the increasing expression profile of *crt1* (log_2_(fold change): 6 h: 5.46; 12 h: 6.47; 24 h: 7.54). It was showed that Crt1 is a lactose permease essential for cellulase induction [68] while Str1 is crucial for pentose utilization and has a role on xylanase induction [47]. Thus, other proteins encoded by these upregulated genes can be potential candidates to sense and transduce signals related to biomass deconstruction and thus potential targets for strain improvement.

We identified 43 groups of candidate orthologues genes between *A. niger* and *T. reesei* that have at least one gene upregulated in our dataset (Additional file 15). We observed contrasting expression profiles among the genes in these groups of orthologues. For instance, the pair of predicted orthologues genes An03g01620 in *A. niger* and 33630 in *T. reesei*, both are annotated as sugar transporters, the former as a putative high affinity glucose transporter (AspGD) and the latter as a xylose transporter [69]; only the *T. reesei* gene was found induced by SEB, at all time points (Additional file 15). Another example is found with the pair An18g01700 (*A. niger*) and 85254 (*T. reesei*). An18g01700 is the predicted orthologue of the *Saccharomyces cerevisiae* Hxt8-coding gene and we found it among the SEB induced genes; however, the respective *T. reesei* orthologue did not respond to SEB. These observations strengthen the idea that the two strains have different mechanisms for lignocellulosic biomass degradation.

We also identified several additional upregulated transcripts in both fungal strains that were described as transporters of compounds such as ions, amino acids, and peptides (81 in *A. niger* and 68 in *T. reesei*, Additional file 16). It is worth noting that iron and siderophore transport genes were induced, with considerably larger fold changes in *T. reesei* (Additional file 16). The iron cation is required in Fenton reactions that produce hydroxyl radicals, which can help disrupt lignocellulose [70]. Alternatively, inducing iron transport can be trigger by the depletion of this cation as it binds to cellulose [41]. These observations highlight the fact that not only sugar-degradation and assimilation metabolic pathways are modulated during biomass degradation.

### Transcription factors

Expression and secretion of CAZymes and other enzymes and proteins required for biomass deconstruction are energy-demanding processes. Thus, they will only be produced at a higher quantity when fungi sense the polysaccharides from available plant biomass, and no alternative carbon sources are available, consequently minimizing energy demands. Although the complete mechanisms for the regulation of this process remains unclear, it is known that it is driven by transcription factors (TFs).

In this section we paid attention to TF genes as described by Pel et al. [62] for *A. niger* and by the Schmoll et al. [71] for *T. reesei*. In total, 152 upregulated TFs were found in *A. niger* (Additional file 17) and 52 in *T. reesei* (Additional file 18), most of them not been functionally characterized so far. Twenty-three TF genes have been previously described as being related to biomass deconstruction (Table 4), for 13 of these we found candidate orthologues in both fungal strains, four were only present in *A. niger*, five only in *T. reesei* and one was not found in either strains (*galR*, described in *A. nidulans* [72]). Only two out of the 13 TF genes found in both fungi were induced by SEB (*xlyR/xyr1* and *malR*), but they had distinct expression patterns. In *A. niger*, the expression of these two regulators was upregulated at all time points, similar to the expression profiles of the CAZymes (Fig. 4), *i e.,* decreasing the expression during the time course (Table 4). In *T. reesei*, the expression of CAZymes was detected since the early time points and raised up to 24 h and the major activator *xyr1* was only found induced at 24 h (log_2_(fold change): 1.65). *Xyr1* was expressed at a higher level when *T. reesei* grown on SEB than fructose, during the entire time course; however, at early time point the differences in expression values was below our chosen threshold (log_2_(fold change): 6 h: 0.49; 12 h: 0.99). The TF *malR* has been described as being induced by maltose and as responsible for the expression of the maltose-utilizing gene cluster[73]; why this regulator is induced in both fungi is intriguing, as the SEB did not have a significant quantity of this sugar [74, 75].

**Table 4:** Main proteins involved in biomass deconstruction regulation and their expression in *A. niger* N402 and *T. reesei* RUT-C30 transcriptomes

Gene	ID *A. niger*	ID *T. reesei*	*A. niger* up regulation (Log_2_(FC))	*T. reesei* up regulation (Log_2_(FC))	Function	References
*xlnR/xyr1*	An15g05810	98788	6h (2.59), 12h (2.27), 24h (2.22)	24h (1.65)	Major activator for cellulase and xylanase genes	[123, 124]
*creA/cre1*	An02g03830	23706	6h (1.18), 12h (1.50)	-	Key regulator of CCR, suppressing the transcription of cellulase and xylanases genes	[125, 126]
*araR*	An04g08600		-	-	Control the L-arabinose catabolic pathway and the expression of genes encoding to enzymes related to arabinose metabolism	[127]
*amyR*	An04g06910	42772	-	-	Regulator of starch degradation and controls the production of D-glucose and D-galactose releasing enzymes	[128]
*ace1*	An16g02040	122363	-	-	Repressor of cellulase and xylanase gene expression	[129]
*ace2*	-	32395	-	-	Activator of cellulase and xylanase gene expression	[130]
*ace3*	-	98455	-	12h (1.27), 24h (2.11)	Activator of cellulase and xylanse gene expression and has an unclear role on *xyr1* expression	[131]
*inuR*	An15g00300	-	24h (1.42)	-	Induce the expression of inulinolytic genes	[80]
*bglR*	-	91236	-	-	Activator of specific β-glucosidase genes expression	[132]
*clr-1*	An05g00020	68701	-	-	The orthologue in *Neurospora crassa* has positive role on cellulase production	[133]
*clr-2/clrB/ manR*	An12g01870	76250	-	6h (2.13), 12h (2.87), 24h (3.40)	The orthologue in *Neurospora crassa* has positive role on cellulase production. In *A. oryzae*, is a regulator of genes enconding mannan and cellulose degrading enzymes	[133, 134]
*galX*	An16g01640	-	24h (1.11)	-	Regulates the D-galactose oxido-reductive pathway	[135]
*galR*	-	-		-	Regulates the D-galactose oxido-reductive pathway in *A. nidulans*	[72]
*hap-2/hapB*	An15g03650	93466	-	-	Form the protein complex Hap2/3/5 that represents a positive transcriptional activator that binds to the promoter region of cellobiohydrolase gene	[136]
*hap-3/hapC*	An01g02620	24298	-	-	Form the protein complex Hap2/3/5 that represents a positive transcriptional activator that binds to the promoter region of cellobiohydrolase gene	[136]
*hap-5/hapE*	-	125434	-	-	Form the protein complex Hap2/3/5 that represents a positive transcriptional activator that binds to the promoter region of cellobiohydrolase gene	[136]
*rhaR*	An13g00910	101004	6h (1.75), 12h (1.18), 24h (1.58)	-	Activator of pectinase-encoding genes	[77]
*malR*	An11g04160	133808	6h (2.41), 12h (2.20), 24h (2.02)	6h (1.17), 12h (1.54)	Controls the expression of maltose utilizing genes	[73]
*xpp1*		101064	-	-	Regulator of xylanases but not cellulases genes	[137]
*ron1/xprG*	An16g09130	112541	6h (1.24)	-	Key activator of the GlcNAc gene cluster and essential for chitin catabolism	[138]
*clbR*	An12g04970	-	-	-	Regulator involved in cellobiose and cellulose induction	[139]
*pacC/pac1*	An02g07890	95791	-	6h (2.28), 12h (2.15), 24h (1.94)	pH transcription regulator, also involved in cellulose gene expression	[140, 141]
*areA*	An12g08960	140814	-	-	A global nitrogen metabolism regulator, with influence on cellulase production	[142]

We also found that the transcriptional activator *rhaR,* involved in pectin degradation, was induced in *A. niger*. The sugarcane cell wall has a low proportion of pectin (~10%), and while most of that should be solubilized and washed-out during the steam-explosion used as pre-treatment [75], some can remain available and used by the fungi. Particularly, *A. niger* is known as a good producer of pectinase [76] and in a previous study we observed that several pectinases were secreted when A*. niger* grew on SEB [44]. In our transcriptomics dataset we found 35 pectin-deconstruction genes induced in SEB, which are 60.3% of all the genes related to pectin-deconstruction found in the genome of *A. niger* [76] (Additional file 4); *rhaR*, the pectinase transcriptional activator [77], was found induced at all time points (An13g00910; log_2_(fold change): 6 h: 1.75; 12 h: 1.18; 24 h: 1.58) (Table 4). The predicted *T. reesei rhaR*-orthologue (101004) was not induced in SEB and few pectinases were upregulated in this carbon source (Additional file 5).

The carbon catabolite repressor *creA* in *A. niger* (An02g03830) was upregulated at the initial time points in SEB (6 and 12 h). In *A. nidulans* CreA mediates the repression of *xlnR*, and in *A. niger* the expression of *creA* is associated to the repression of cellulases and hemicellulases, including the major activator *xlnR* (An15g05810) [78, 79]. Surprisingly, we also found *xlnR* to be induced since the earliest time point, suggesting a more complex regulatory relationship between *creA* and *xlnR* in *A. niger*. In *T. reesei*, Ace3 (98455) positively regulates the expression of cellulases, and we found this gene induced at 12 h and 24 h. *xpp1* (101064), a positive regulator of xylanase expression, was downregulated at the early time points, with no significant difference in expression between SEB and fructose at 24 h (log_2_(fold change): 6 h, -1.47; 12 h, -2.53; 24 h, 0.02). *Cre1* (23706) was slightly downregulated, with a fold change smaller than our selection criterion. In relation to other predicted TFs orthologues, we found four pairs of orthologues upregulated in both strains (An18g05600/ 77124, An16g06780/ 67125, An11g03890/ 39977, An09g01870/ 93861), and despite this interesting result, their functions remain to be elucidated.

The results presented above on TF genes, relied on previous identification of TFs [62, 71]. We decided to look for the genes that have protein domains frequently associated with transcription factor activity but that have not been identified as such in previous studies. Several induced putative TF genes were identified in both fungi with domain frequently associated to transcription factors (Additional file 19), such as fungal specific transcription factor domain (PF04082, PF11951), bZIP (PF00170), Fungal Zn(2)-Cys(6) binuclear cluster domain (PF00172). In this source, we identified *inuR* and *rhaR* that have not been described by Pel et al. [62], but that were characterized by Yuan et al. [80] and Gruben et al. [77], respectively. Therefore, further studies are necessary to elucidate their role in the regulation of lignocellulose degradation.

Although *A. niger* and *T. reesei* share some TFs of recognized importance in lignocellulose degradation (such as XlnR/Xyr1 and CreA/ Cre1), they have clear differences concerning TF content and in their regulation of these genes. This clearly suggests that these two strains have different regulatory circuits at play for the degradation of complex biomass sources. A better understanding of these regulatory circuits is required to improve the genetic manipulation of these organisms, so that new strains can be generated with enhanced enzymatic potential.

### Other genes of interest

The process of biomass saccharification releases different sugars to the environment. This leads to the induction of several genes in an array of sugar metabolic pathways, for instance in the arabinose and xylose metabolic pathways, the genes coding for D-xylose/xylulose reductase, xylitol dehydrogenase, xylulokinase and arabinitol dehydrogenase were induced in almost all time points (Additional file 20). Also of interest, we found that from the eight hydrophobins predicted in the *A. niger* genome [81], seven were found as being upregulated induced in SEB and the profile expression of one of them (*hfbA,* An03g02400) was validated by RT-qPCR (Additional file 8). According to Schmoll et al. [71], *T. reesei* QM6a genome has 10 genes coding hydrophobins, we could identify nine of these in the genome of *T. reesei* RUT C30, and only two (125146 and 89290) were induced by SEB (Additional file 20). Hydrophobins are small fungal proteins (≤ 20 kDa) able to adsorb the hydrophobic surfaces and mediate the interaction between the fungi and the medium through amphipathic layers [82]. They reduce the substrate surface tension in which the fungus is growing [82] and are involved in biofilm formation over the lignocellulosic biomass, as observed in *A. nidulans* grown on SEB [83]. Furthermore, hydrophobins are potential candidates for *T. reesei* and *A. niger* adhesion at the bagasse’s cell wall.

Moreover, swollenin (*swo1*, 104220) and *cip1* (121449), which also have role in biomass degradation [84, 85], showed high RPKM values and log_2_(fold change) in *T. reesei* (Additional file 20), especially after 24 h of growth. The high expression of these genes was also validated by RT-qPCR (Additional file 9). It was demonstrated that swollenin can disrupt the cellulose structure by cleaving hydrogen bonds between the lignocellulose polysaccharides [86]. Swo1 displays endoglucanase activity being able to hydrolyze β-1,4 linkages in β-glucan and carboxymethylcellulose (CMC) [85]. It is not yet clear what the Cip1 function is, but this protein has a secretion signal and a CBM1 domain [87]. *Cip1* presented an increasing expression along the time series (log_2_(fold change): 6 h: 3.86; 12 h: 6.24; 24 h: 9.26) and was also strongly induced in wheat straw [40] and sophorose [88]. In these two studies the expression of *cip1* was similar to the profiles of endoglucanases and of the cellobiohydrolase *cbh1*, suggesting a potential of Cip1 in cellulose degradation. Interestingly, *A. niger* does not contain either of these genes, which reiterates the different strategies used by both fungi during biomass degradation.

Recently, a new class of enzymes called PADs (Pro-oxidant, Antioxidant, and Detoxification enzymes) has been drawing attention due to its putative participation in lignocellulose breakdown. PADs are detoxifying and lignin/phenol modification enzymes, such as catalase (CAT), superoxide dismutase (SOD), peroxidase, cytochrome P450 (p450), glutathione S-transferase (GST) and aldo-keto reductase (AKR). There are few papers in the literature relating the influence of these enzymes in the biomass saccharification and oxidative stress control [89–95]. We found that several genes coding for PADs were induced by SEB; in *T. reesei,* 1 CAT, 6 GSTs and 7 AKRs, while 2 CATs, 2 SODs, 10 GSTs, 4 peroxidases and 12 AKRs were induced in *A. niger*, some of them with high fold change values, such as An08g09150 and An07g00030 (Additional file 20). These enzymes could be acting on the oxidative control of the highly reactive ROS generated by the oxidative degradation driven by LPMOs (AA9) and other oxidative enzymes (AA3; AA6; AA7). And according to the high lignin proportion of steam-exploded bagasse composition (34%,), the peroxidases and aldo-keto reductases might be participating in the removal of lignin fraction, which can be as high as 34% in SEB [96], enabling access to the cellulose by conventional GHs, as suggested previously [89, 97–99]. The role of these enzymes on biomass deconstruction is poorly explored in ascomycetes, but their upregulation in *T. reesei* RUT-C30 and *A. niger* N402 during the growth in SEB suggests that they can be acting (directly or indirectly) in this process.

**Fig. 6:**
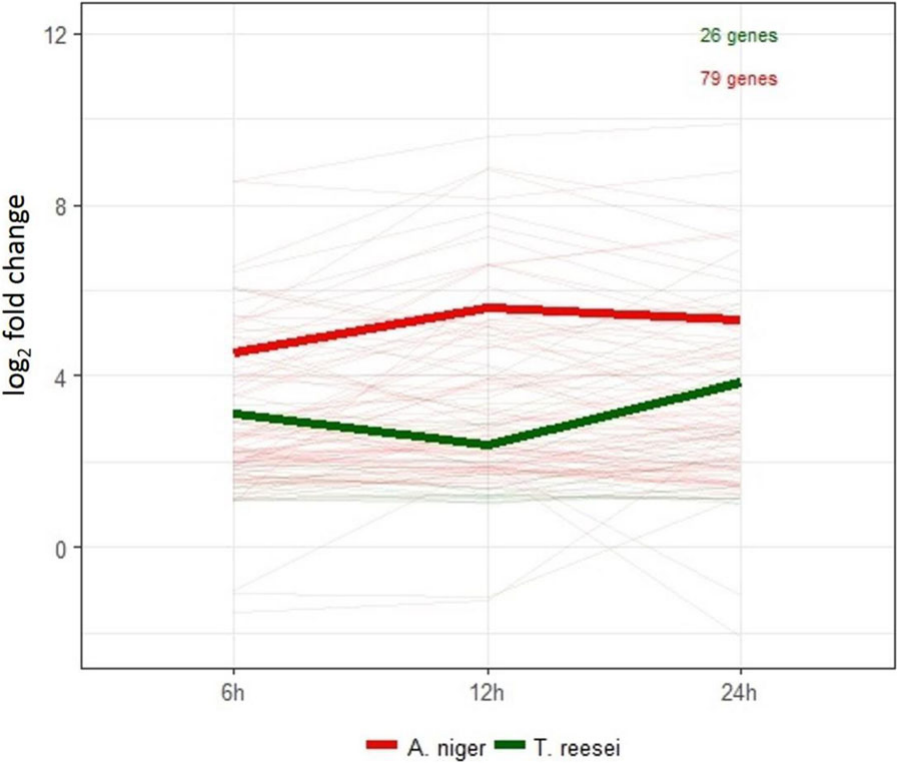
Expression profile of genes coding unknown proteins with peptide signal predicted upregulated in *A. niger* N402 and *T. reesei* RUT-C30 transcriptomes. Thinner lines represent the individual gene expression (log_2_(fold change)) for each fungus and the thicker ones the average of these values. Only genes upregulated in all time points (6, 12 and 24 h) were considered

Finally, we turn our attention to potentially new genes with important roles in biomass degradation. We focused on genes that have signal peptide cleavage sites, that could be secreted, and that have no reported function either at AspGD or JGI databases, and that do not have significant hits to protein domains. In *A. niger,* 146 genes were upregulated out of which 76 were induced in all time points (Additional file 21), some of them with high fold changes, being among the 20 most induced genes (Additional file 11). They showed an expression profile (Fig. 6) that is different from CAZymes (Fig. 4) and transporters (Fig. 5). In *T. reesei*, 66 genes of unknown function were identified as being upregulated, 25 of them in all growth conditions (Additional file 21). The high expression of these genes suggests that they can have a role on the saccharification metabolism, and are interesting targets for follow up studies.

## Conclusion

This study describes a comparative transcriptome analysis of *T. reesei* RUT-C30 and *A. niger* N402 during growth on steam-exploded sugarcane bagasse as carbon source. A set of CAZymes, such as cellulases, hemicellulases and oxidative enzymes was expressed by both fungal strains, but their gene expression profiles was opposite to each other. During the time series evaluated in this work, *T. reesei* had an increasing activation of its CAZymes while *A. niger* had a faster transcriptional response, upregulating the most of them at 6 h and decreasing their transcription after that. Our findings revealed both strains upregulated several genes, which code for putative transporters of sugars and other small molecules, transcription factors, proteins of unknown functions and PAD enzymes. These proteins can be players in biomass degradation and deserve further studies to verify their role. Altogether, the data show that *A. niger* N402 and *T. reesei* RUT-C30 have differences in genes related to lignocellulose breakdown content and also in their expression profile. This knowledge can be exploited for the rational design of enzymatic cocktails and 2G ethanol production improvement.

## Methods

### Fungal strains and culture conditions

The fungi *Trichoderma reesei* RUT-C30 (ATCC 56765) and *Aspergillus niger* N402 (ATCC 64974) were kindly provided by Dr. Bernhard Seiboth and Dr. David Archer, respectively. As mentioned before, *T. reesei* RUT-C30 strain was obtained after three random mutagenesis (with UV light and N-nitroguanidine) of the wild type QM6a [17]. *A. niger* N402 strain was produced in 1983 from N400 by two rounds of UV mutagenesis (personal communication, Dr Fons Debets, University of Wageningen). Both strains were kept in silica gel desiccant with 7% milk (w/v) at 4°C [100]. Fungi were grown on basic culture medium (BCM) [44] with a predetermined concentration of carbon source according to our experimental conditions.

For cultivation in medium with SEB, the mycelia grown on the BCM were filtered, washed twice with sterile distilled water in order to eliminate any residual sugar and then transferred to fresh BCM deprived of 0.05% yeast extract but with 0.5% of SEB (w/v) as carbon source. SEB was treated as described by De Souza et al. [34], and exhaustively washed with deionized water until reducing sugars were not detected by DNS [101]. SEB was completely dry at 60°C for several days and sifted in a 600 μm industrial sieve. Cellulose, hemicellulose and lignin proportion made up 47, 9 and 34 % of the SEB, respectively [96]. All media were sterilized in an autoclave for 20 min at 121 °C before using.

### Substrate-based induction conditions

For RNA sequencing (RNA-seq) and quantitative reverse-transcription PCR (RT-qPCR) experiments, *T. reesei* and *A. niger* spores were first cultivated in potato dextrose agar plate for 7-10 days at 29°C and 30°C, respectively, and harvested by adding 1 mL of sterile distilled water. The spore suspensions were inoculated to a final concentration of 1 × 10^6^ spores per 30 mL of BCM culture in 250 mL flasks. *T. reesei* and *A. niger* spores were grown on BCM with 1% fructose (w/v) as the carbon source at 29°C and 30°C, respectively, for 24 hours (h) (*A. niger*) or 48 h (*T. reesei*) in a rotary shaker with agitation of 200 rpm. After, the mycelia were transferred to 0.5% SEB (w/v) as the carbon source for 6, 12 and 24 h, and to 1% fructose (w/v) for 24 h for the RNA-seq experiment and 6, 12 and 24 h for the RT-qPCR. The RNA-seq and RT-qPCR experiments were carried out in duplicate and triplicate, respectively. Fructose was used as control condition in all experiments because it is an inert sugar, which neither induces nor suppresses overall expression of lignocellulolytic enzymes [102]. For the growth of *T. reesei*, the cultures were maintained in constant light, as this influences positively cellulase gene expression [103]. Mycelia were harvested by filtration through Whatman grade 1 filters (GE Healthcare, Grandview Blvd. Waukesha, WI, USA), washed thoroughly with sterile water and immediately ground into powder in liquid nitrogen. Aliquots of 100 mg ground mycelia were harvested and kept at -80 °C until the RNA extraction.

### RNA extraction and RNA-seq

Total RNA from aliquots was extracted using the RNeasy Plant Mini kit (Qiagen) according to manufacturer’s instructions. RNA concentration was determined in a Nanodrop 2100c (Thermo Fisher Scientific, Waltham, MA, USA) and the integrity was evaluated in a 2100 Bioanalyzer (Agilent Technologies, Santa Clara, CA, USA) using the Agilent RNA 6000 Nano kit (Agilent Technologies, Santa Clara, CA, USA). Total RNA from two biological replicates per condition (SEB or fructose) from both fungi was used as input for the TruSeq RNA Sample Prep v2 kit (Illumina, San Diego, CA, USA) and the libraries were sequenced at the USC Epigenome Center (CA, USA) using the Illumina® HiSeq2000 system, producing single-end reads of 50 bp.

### RNA-seq analysis

More than 30 million 50 bp single-end reads were obtained for both fungi after sequencing. First, these reads were size-filtered (minimum of 40 bp) and selected by quality (Q > 20) by AlienTrimmer software [104]. Filtered reads were mapped to the *T. reesei* RUT-C30 v1.0 genome available in the JGI Genome Portal (9,852 gene models, [105, 106]) and to *A. niger* CBS 513.88 genome from Aspergillus Genome Database AspGD (14,165 gene models, [62, 107]), using TopHat2 [108]. Saturation of exon-exon junctions at different sub-sampling fractions of the total number of reads was assessed to verify proper sequencing coverage, using RseQC [109]. Mapped reads were counted with the featureCounts function from the Rsubread v1.12.6 package [110] in R v3.0.2 [111]. Read counts were normalized using TMM [112]. Differentially expressed genes (DEGs) were identified with NOISeqBIO v2.8.0 [113] with the following parameters k = 0.5, nclust = 15, r = 20, lc = 1, filter = 1, cpm = 1; in order to minimize false positives and false negatives, the threshold value for significance q = 0. 95 was used, which is equivalent to a FDR cutoff of 0.05. Only DEGs with two-fold change cutoff (SEB versus fructose) were considered, i.e., log_2_-fold change ≥ 1 (upregulated) or ≤ - 1 (downregulated). RPKM values were computed after TMM normalization for comparison and visualization of the transcriptional profiles of both fungi.

Further, upregulated genes were grouped into different categories according to functional information. CAZymes categories includes all GH, CE, PL, AA genes predicted by CAZy [53], but also available at the JGI and AspGD databases, only excluding the CE10 family that has been associated with non-carbohydrate substrates hydrolysis. Glycosyl transferases (GTs) were also removed from consideration as they work on fungal cell wall remodeling. Additionally, CAZymes and their annotations were retrieved from Benoit et al. [114] for *A. niger* and Häkkinen et al. [43] for *T. reesei*. Genes encoding CAZymes predicted by the dbCAN pipeline [115] (parameters: e-value < 1.00E-17; coverage > 0.45), but not found in JGI, AspGD database or in the previously mentioned publications, were included as well and labeled as putative CAZymes. Putative sugar transporter proteins (mainly, MFS and ABC transporters) were labeled as “transporters” and those related to ion, amino acids and drugs as “other transporters”. The number of transmembrane helices was assessed by the TMHMM Server v2.0 [116]. Transcription factors were annotated following Pel et al. [62] and the Schmoll et al. [71] for *A. niger* and *T. reesei*, respectively. New candidate transcription factors were identified following the strategy proposed in Perez-Rodriguez et al. [117], with a set of updated classification rules (Renato Santos et al., unpublished results). For genes encoding proteins with no functional annotation in the JGI or AspGD databases, PFAM domains were searched using Motif Search tool [118]. The peptide signal was predicted by SignalP v4.1 [119] and it was considered as proteins of unknown function the ones containing PFAM domains with e-value > 1.00E-10.

Gene Ontology (GO) annotation was obtained from JGI and AspGD and the enrichment analysis was performed with the Cytoscape’s plugin BINGO 3.0.2, using the hypergeometric test and adjusting p-values for multiple testing with the Benjamini & Hochberg's false discovery rate (FDR) method (p ≤ 0.05). Orthologues between *T. reesei* and *A. niger* were predicted by applying the OrthoMCL pipeline [120]. Briefly, protein sequences were downloaded from JGI and AspGD databases, respectively. The all-vs-all BLAST similarity search was performed keeping hits with e-value ≤ 10^-5^. Orthologues identification groups was carried out with an inflation value of 1.5 (parameter of cluster tightness) [120] and a homology percent match threshold of 60%.

### RT-qPCR analysis of selected genes

After RNA-seq analysis, 9 DEGs from each fungal that were among the most upregulated genes, including CAZymes, transporters and other genes of interest were chosen for validation by RT-qPCR. Briefly, extracted RNA was treated with TURBO DNA-free kit (Applied Biosystems, Life Technologies) for depletion of any residual genomic DNA. SuperScript™ II Reverse Transcriptase (Invitrogen, Life Technologies) was used to synthesize cDNA from 400 ng of total RNA according to the manufacturer’s instructions. Specific primers for each transcript were designed to amplify a fragment between 80 bp and 150 bp (Additional file 22), a first round of end-point PCR was performed with these primers and the products were separated by agarose gel electrophoresis and purified with PureLink Quick Gel Extraction kit (Invitrogen, Life Technologies). Purified PCR products were sequenced in 3500xL Genetic Analyzer (Applied Biosystems, Life Technologies), using BigDye® Terminator v3.1 Cycle Sequencing (Applied Biosystems, Life Technologies) to confirm the amplicon sequences identity. The amplicons were subjected to a 10-fold serial dilution (from 10^-1^ to 10^-8^) and used to construct a standard curve. Real-time PCR reactions were performed along with the five best points of the standard curve and the cDNA samples from the experiments (see above), to keep the same conditions for standards and experimental samples (relative standard curve method). The reactions were performed with 1.0 μL of cDNA or point dilution, 5.0 μL of SYBR® Green PCR Master Mix (Applied Biosystems, Life Technologies) and 2 pmol of each primer, in a final volume of 10 μL. All RT-qPCR reactions were carried out in ViiA 7 Real Time PCR system (Applied Biosystems, Life Technologies) using the following amplification conditions: activation for 10 minutes at 95°C followed by 40 cycles of denaturation (15 seconds at 95°C), annealing and extension (1 minute at 60°C). *Act* and *sar1* genes were chosen for data normalization of *T. reesei* and *A. niger* gene expression, respectively [121, 122]. Only primer pairs with amplification efficiencies ≥ 85% and correlation coefficient (r2) ≥ 0.99 were considered for analysis. Data acquisition and melting curve analysis were performed in ViiA RUO software (Applied Biosystems, Life Technologies).

## Additional files

Additional file 1: Result of RNA sequencing for *A. niger* N402 and *T. reesei* RUT-C30 grown on sugarcane SEB and fructose. (XLSX 12 kb)

Additional file 2: All DEGs found in the transcriptomes of *A. niger* N402 and *T. reesei* RUT-C30 grown on sugarcane SEB. (XLSX 1286 kb)

Additional file 3: Gene ontology enrichment analysis. (XLSX 17 kb)

Additional file 4: Differentially expressed CAZymes upregulated in the *A. niger* N402 transcriptome. (XLSX 1000 kb)

Additional file 5: Differentially expressed CAZymes upregulated in the *T. reesei* RUT-C30 transcriptome. (XLSX 1074 kb)

Additional file 6: Predicted orthologues of genes coding CAZymes upregulated in both *A. niger* N402 and *T. reesei* RUT-C30 transcriptomes. (XLSX 14 kb)

Additional file 7: Transcriptional profile of main CAZy families related to lignocellulose degradation. (XLSX 444 kb)

Additional file 8: Validation of *A. niger* N402 RNA-seq data by RT-qPCR. (XLSX 355 kb)

Additional file 9: Validation of *T. reesei* RUT-C30 RNA-seq data by RT-qPCR. (XLSX 320 kb)

Additional file 10: Expression of the twenty most upregulated genes in *T. reesei* RUT-C30 transcriptome. (XLSX 26 kb)

Additional file 11: Expression of the twenty most upregulated genes in *A. niger* N402 transcriptome. (XLSX 30 kb)

Additional file 12: Putative new CAZymes of *A. niger* N402 and *T. reesei* RUT-C30 differentially expressed in their transcriptomes and identified by dbCAN. (XLSX 16 kb)

Additional file 13: Known and putative transporters upregulated in *A. niger* N402 transcriptome. (XLSX 41 kb)

Additional file 14: Known and putative transporters upregulated in *T. reesei* RUT-C30 transcriptome. (XLSX 26 kb)

Additional file 15: Predicted orthologue genes which code upregulated transporters in both *A. niger* N402 and *T. reesei* RUT-C30 transcriptomes. (XLSX 21 kb)

Additional file 16: Other putative upregulated transporters in both *A. niger* N402 and *T. reesei* RUT-C30 transcriptomes. (XLSX 196 kb)

Additional file 17: Known upregulated transcription factors in *A. niger* N402 transcriptome. (XLSX 36 kb)

Additional file 18: Known upregulated transcription factors in *T. reesei* RUT-C30 transcriptome. (XLSX 19 kb)

Additional file 19: Upregulated transcription factor-likes in both *A. niger* N402 and *T. reesei* RUT-C30 transcriptomes. (XLSX 20 kb)

Additional file 20: Interesting upregulated genes in *A. niger* N402 and *T. reesei* RUT-C30 transcriptomes. (XLSX 33 kb)

Additional file 21: Unknown proteins with predicted signal peptide whose genes were upregulated in *A. niger* N402 and *T. reesei* RUT-C30 transcriptomes. (XLSX 6133 kb)

Additional file 22: Primers used in the RT-qPCR analysis. (XLSX 13 kb)
